# Changes in neuronal activity across the mouse ventromedial nucleus of the hypothalamus in response to low glucose: Evaluation using an extracellular multi‐electrode array approach

**DOI:** 10.1111/jne.12824

**Published:** 2020-02-23

**Authors:** Lydia Hanna, Tristan J. Kawalek, Craig Beall, Kate L. J. Ellacott

**Affiliations:** ^1^ Reading School of Pharmacy University of Reading Reading UK; ^2^ Institute of Biomedical & Clinical Sciences University of Exeter Medical School Exeter UK; ^3^ Present address: Department of Biological Sciences Centre for Biomedical Sciences Royal Holloway University of London Egham UK

**Keywords:** electrophysiology, glucose‐sensing, hypothalamus, multi‐electrode array, ventromedial nucleus

## Abstract

The hypothalamic ventromedial nucleus (VMN) is involved in maintaining systemic glucose homeostasis. Neurophysiological studies in rodent brain slices have identified populations of VMN glucose‐sensing neurones: glucose‐excited (GE) neurones, cells which increased their firing rate in response to increases in glucose concentration, and glucose‐inhibited (GI) neurones, which show a reduced firing frequency in response to increasing glucose concentrations. To date, most slice electrophysiological studies characterising VMN glucose‐sensing neurones in rodents have utilised the patch clamp technique. Multi‐electrode arrays (MEAs) are a state‐of‐the‐art electrophysiological tool enabling the electrical activity of many cells to be recorded across multiple electrode sites (channels) simultaneously. We used a perforated MEA (pMEA) system to evaluate electrical activity changes across the dorsal‐ventral extent of the mouse VMN region in response to alterations in glucose concentration. Because intrinsic (ie, direct postsynaptic sensing) and extrinsic (ie, presynaptically modulated) glucosensation were not discriminated, we use the terminology ‘GE/presynaptically excited by an increase (PER)’ and ‘GI/presynaptically excited by a decrease (PED)’ in the present study to describe responsiveness to changes in extracellular glucose across the mouse VMN. We observed that 15%‐60% of channels were GE/PER, whereas 2%‐7% were GI/PED channels. Within the dorsomedial portion of the VMN (DM‐VMN), significantly more channels were GE/PER compared to the ventrolateral portion of the VMN (VL‐VMN). However, GE/PER channels within the VL‐VMN showed a significantly higher basal firing rate in 2.5 mmol l^‐1^ glucose than DM‐VMN GE/PER channels. No significant difference in the distribution of GI/PED channels was observed between the VMN subregions. The results of the present study demonstrate the utility of the pMEA approach for evaluating glucose responsivity across the mouse VMN. pMEA studies could be used to refine our understanding of other neuroendocrine systems by examining population level changes in electrical activity across brain nuclei, thus providing key functional neuroanatomical information to complement and inform the design of single‐cell neurophysiological studies.

## 1 INTRODUCTION

The brain is critical for maintaining systemic glucose homeostasis, in part by direct sensing of changes in glucose levels. Neurophysiological studies in rodent brain slices have identified glucose‐sensing neurones that change their firing frequency in response to alterations in local glucose levels. Glucose‐sensing neurones are broadly subdivided into two groups: glucose‐excited (GE) and glucose‐inhibited (GI). GE neurones show enhanced firing activity in response to higher ambient glucose concentrations, whereas the reverse is true for GI neurones. The hypothalamus is a key brain region mediating glucose homeostasis and glucose‐sensing neurones have been identified in several hypothalamic nuclei: the arcuate nucleus (ARC), lateral hypothalamus, paraventricular nucleus and ventromedial nucleus (VMN).^1^ Microdialysis studies in rats have shown that glucose concentrations in the ventromedial region of the hypothalamus (VMH, which comprises the ARC and VMN) are approximately 20%‐30% that of peripheral euglycaemic (‘normal’) levels: approximately 1.0‐2.5 mmol L^‐1^ (VMH) and 5.5 mmol L^‐1^ (blood). Within the VMH, these can reach 5 mmol L^‐1^ during hyperglycaemia and 0.2 mmol L^‐1^ during hypoglycaemia.^2^, ^3^, ^4^ Therefore, the glucose concentrations and time courses used in the present study model pathophysiologically relevant variations seen in people with diabetes.

The VMN is one of the best studied hypothalamic nuclei with respect to glucose‐sensing neurones, with both GE and GI neurones observed.^1^, ^5^ VMN glucose‐sensing neurones play an important role in detecting and reacting to glucose deficit, regulating both the counter regulatory response to hypoglycaemia and glucoprivic feeding.^6^ GE and GI neurones are defined as being directly/intrinsically responsive to alterations in extracellular glucose levels.^7^ Indeed, in the presence of the voltage‐gated sodium channel blocker, tetrodotoxin (TTX), direct postsynaptic changes (ie, in the resting membrane potential) are seen in both GE and GI neurones in various brain areas.^8^, ^9^ Three subtypes of non‐intrinsic, glucose‐sensing VMN neurones have also been described, which are modulated by presynaptic glutamatergic inputs. These extrinsically glucose‐sensing neurones include PED neurones, which are presynaptically excited by a decrease (PED) in extracellular glucose levels (from 2.5 to 0.1 mmol L^‐1^ glucose), and PER and PIR neurones, which are either presynaptically excited (PER) or inhibited (PIR) by an increase in extracellular glucose levels (from 2.5 to 5‐10 mmol L^‐1^ glucose), respectively.^10^

Unlike other hypothalamic nuclei where the neuropeptide/neurotransmitter phenotype of glucose‐sensing neurones has already been identified,^1^ this is less clear for the VMN. To date, most of the ex vivo slice electrophysiological studies characterising VMN glucose‐sensing neurones in rodents have utilised the patch clamp technique, which enables real‐time detailed interrogation of the electrical properties of individual glucose‐sensing neurones. However, because only a single cell can be studied at a given time, and given that intrinsic GE and GI neurones are estimated to together comprise only approximately 20% of the VMN neuronal population,^11^ this makes the patch clamp technique both time‐consuming and of relatively low yield. Furthermore, because few glucose‐sensing neurones can be recorded per brain slice, the investigator, who, in this circumstance, is ultimately searching for specialised glucose‐sensing neurones, is potentially introducing unintentional anatomical selection bias in their recordings by targeting parts of the VMN known to be enriched in these cells.

Multi‐electrode arrays (MEAs) comprise an electrophysiological tool enabling the electrical activity of large neuronal groups to be simultaneously recorded across multiple electrode sites (channels). This powerful neurophysiological extracellular recording technique is used both in vivo and ex vivo.^12^ An advantage of this method compared to others is the high spatial and temporal resolution that it offers, enabling the functional investigation and mapping of complex and heterogeneous nuclei, such as the VMN.^13^, ^14^ Each MEA electrode site can record the electrical activity from a population of neurones: referred to as multi‐unit activity (MUA). Use of conventional spike sorting methods allows the activity of single cells (single‐unit activity [SUA]) to be discriminated from the MUA.^15^ We have previously employed perforated MEA (pMEA) technology for ex vivo hypothalamic recordings from mouse brain slices 16 because of the improved slice perfusion rate, long‐term recording stability and high signal‐to‐noise ratio.^17^ In the present study, we have used an equivalent pMEA extracellular recording approach in ex vivo adult mouse brain slices aiming to objectively evaluate changes in neuronal activity across the dorsal‐ventral extent of the medial portion of the VMN in response to changes in extracellular glucose concentration. To our knowledge, this is the first study utilising the MEA system to evaluate glucose responsivity across the mouse VMN neural network.

## 2 MATERIALS AND METHODS

### 2.1 Animals

Male 10‐13 week‐old C57BL/6J mice bred at the University of Exeter were used for these studies (n = 5, 1‐2 VMN‐containing slices per animal). Mice were group housed under a 12:12 hour light/dark cycle at 22 ± 2°C, with unlimited access to standard laboratory rodent diet (EURodent diet [5LF2]; LabDiet; PMI Nutrition International, LLC, Brentwood, MO, USA) and water. Studies were performed in accordance with UK Home Office Regulations as defined in the UK Animals (Scientific Procedures) Act 1986 and were approved by the University of Exeter Animal Welfare Ethical Review Board.

### 2.2 Reagents

Unless stated otherwise, all reagents were obtained from Sigma‐Aldrich (Poole, UK) or Fisher Scientific (Loughborough, UK).

### 2.3 Preparation of brain slices

Mice were killed by cervical dislocation followed by decapitation at zeitgeber time (ZT)3 (with lights on defined as ZT0, lights off as ZT12). Brains were rapidly removed and then, along the coronal plane, sectioned using a Leica VT1200 microtome (Leica Microsystems, Wetzlar, Germany) in ice‐cold (~4°C) sucrose‐based solution composed of (in mmol L^‐1^): 189 sucrose, 10 d‐glucose, 26 NaHCO_3_, 3 KCl, 5 MgSO_4_‧7H_2_0, 0.1 CaCl_2_ and 1.25 NaH_2_PO_4_, bubbled with 95% O_2_/5% CO_2_ mixture. Slices (thickness 300 µm) containing the medial portion of the VMN were collected, starting from bregma −1.22 mm up to −2.18 mm 18 (for more details, see Anatomical locations section below). Slices containing the medial‐VMN (one or two per animal) were used for subsequent recordings. Slices were transferred into a Petri dish containing artificial cerebrospinal fluid (aCSF) composed of (in mmol L^‐1^): 10 d‐glucose, 124 NaCl, 3 KCl, 24 NaHCO_3_, 1.25 NaH_2_PO_4_, 1 MgSO_4_‧7H_2_0 and 2 CaCl_2_, continuously bubbled with carbogen. After a 20‐minute rest at room temperature (~22°C), slices were then transferred into a holding chamber containing oxygenated aCSF with physiologically‐relevant brain glucose concentrations (in mmol L^‐1^): 2.5 d‐glucose, 7.5 d‐mannitol, 124 NaCl, 3 KCl, 24 NaHCO_3_, 1.25 NaH2PO_4_, 1 MgSO_4_‧7H_2_0 and 2 CaCl_2_: slices were gradually warmed to ~34°C in the holding chamber and allowed to rest for at least 1 hour.

### 2.4 In vitro multi‐electrode recordings

MEA recordings from acute brain slices were performed as described previously.^16^, ^19^ After 1 hour of rest, a VMN‐containing mouse brain slice was placed recording side down, onto a 60pMEA100/30iR‐Ti‐gr perforated multi‐electrode array (pMEA; Multi Channel Systems, MCS GmbH, Hanover, Germany). These arrays are comprised of 60 electrodes in a 6 × 10 layout (one is a reference electrode), with each electrode 100 µm apart, and with a diameter of 30 µm, covering a total area of approximately 707 μm^2^. Thus, anatomically, a single array can cover the entirety of the mouse VMN.^18^ Contrast illumination (both underneath and above the brain slice) was used to ensure accurate placement of the VMN on the array. To verify VMN positioning over the pMEA electrode sites, images were taken using a GXCAM‐EYE‐5 5MP eyepiece camera (GT Vision, Assington Green, UK) and overlaid. The slice was acclimatised in pre‐warmed oxygenated aCSF containing 2.5 mmol L^‐1^ glucose in the pMEA recording chamber for at least 30 minutes before the recordings began.

To ensure recording stability, slices were held in place by negative pressure provided by vacuum‐generated suction applied via perforations of the MEA and by a weighted slice harp. The pMEA recording chamber was continuously perfused with pre‐warmed (34 ± 1°C) oxygenated aCSF (containing 2.5 mmol L^‐1^ glucose) from above and below at a combined rate of 2.5‐3 mL min^‐1^. Neural signals were acquired using mc_rack software (MCS GmbH) as time‐stamped action potential waveforms using a MEA1060 system and MEA1060UP amplifier (MCS GmbH). Signals were sampled at 50 kHz, high‐pass filtered at 200 Hz (second‐order Butterworth) and multi‐unit spikes crossing a threshold (usually set at −16.5 μV) were extracted for further analysis.

### 2.5 Glucose responsiveness

To investigate the glucose‐sensing capability of VMN neural networks to changing glucose concentrations, recordings were commenced in ‘euglycaemic’ 2.5 mmol L^‐1^ d‐glucose containing aCSF (referred to as 2.5 mmol L^‐1^ glucose). After a 40‐minute baseline recording, slices were then perfused for 15 or 40 minutes in ‘hypoglycaemic’ 0.1 mmol L^‐1^ d‐glucose containing aCSF (referred to as 0.1 mmol L^‐1^ glucose), composed of (in mmol L^‐1^): 124 NaCl, 3 KCl, 24 NaHCO_3_, 1.25 NaH_2_PO_4_, 1 MgSO_4_‧7H_2_0, 0.1 d‐glucose, 9.9 d‐mannitol and 2 CaCl_2_: slices were then allowed to recover in 2.5 mmol L^‐1^ d‐glucose containing aCSF. These glucose concentrations were chosen because they reflect VMN glucose concentrations observed during euglycaemia and hypoglycaemia: approximately 1.0‐2.5 mmol L^‐1^ and 0.2 mmol L^‐1^, respectively.^2^

To investigate the glucose‐sensing capability of VMN neural networks to a less extreme change in glucose concentration (more in line with an overnight fast 10), after a baseline recording in 2.5 mmol L^‐1^ glucose (as above), slices were then perfused for 15 minutes in 0.7 mmol L^‐1^ d‐glucose containing aCSF (referred to as 0.7 mmol L^‐1^ glucose), composed of (in mmol L^‐1^): 124 NaCl, 3 KCl, 24 NaHCO_3_, 1.25 NaH_2_PO_4_, 1 MgSO_4_‧7H_2_0, 0.7 d‐glucose, 9.3 d‐mannitol and 2 CaCl_2_: slices were then allowed to recover in 2.5 mmol L^‐1^ d‐glucose containing aCSF.

No synaptic blockers were used during recordings. Although the voltage‐gated sodium channel blocker TTX has been useful in patch clamp experiments to distinguish between intrinsic and extrinsic glucosensing capabilities of neurones,^8^, ^9^ we could not use this approach during MEA recordings as bath application of TTX abolishes the extracellularly recorded spontaneous action potentials in VMN‐containing brain slices^20^. However, at the end of a subset of experiments, slices were either treated with bath application of the glutamatergic NMDA receptor agonist, NMDA (20 µmol L^‐1^), to confirm maintained cell responsiveness, and/or were followed by application of TTX (1 µmol L^‐1^). Given the strength of the spatial coverage of the pMEA, we chose to focus on the responsiveness of glucose‐sensing neurones to low glucose across the mouse VMN, rather than on intrinsic/extrinsic glucosensation. For conciseness, glucose‐sensing neuronal populations (multi‐unit activity) detected by the pMEA electrodes were termed as: ‘GE/PER’ or ‘GI/PED’ (the latter includes adapting GI channels described previously by others 21, 22).

### 2.6 Statistical analysis

MUA and isolated SUA (ie, from these multi‐unit recordings) were analysed in offline sorter, version 3 (Plexon Inc., Dallas, TX, USA) using principal component analysis‐based spike sorting, as described previously.^16^ Briefly, a distinct cluster of spikes was identified as a single‐unit if confirmed by multivariate analysis of variance *F* statistics and a clear refractory period (>20 ms) in the interspike interval distribution. After spike sorting, the data were then exported to neuroexplorer, version 4 (Nex Technologies, Colorado Springs, CO, USA) and matlab R2016b (MathWorks Inc., Natick, MA, USA) for construction of perievent histograms and further analysis. Glucose‐responsive units were identified as those in which there was a clear peak or trough during the 0.1 or 0.7 mmol L^‐1^ glucose application that exceeded the upper or lower bounds of the 95% confidence limits from the pre‐stimulus spike counts, respectively (10‐second bins). All units that maintained a low firing frequency (< 0.2 Hz) throughout the experiment and/or did not respond to alterations in glucose concentration, including during recovery of glucose levels from 0.1 or 0.7 mmol L^‐1^ back to 2.5 mmol L^‐1^, were considered non‐responsive.

**Figure 1:**
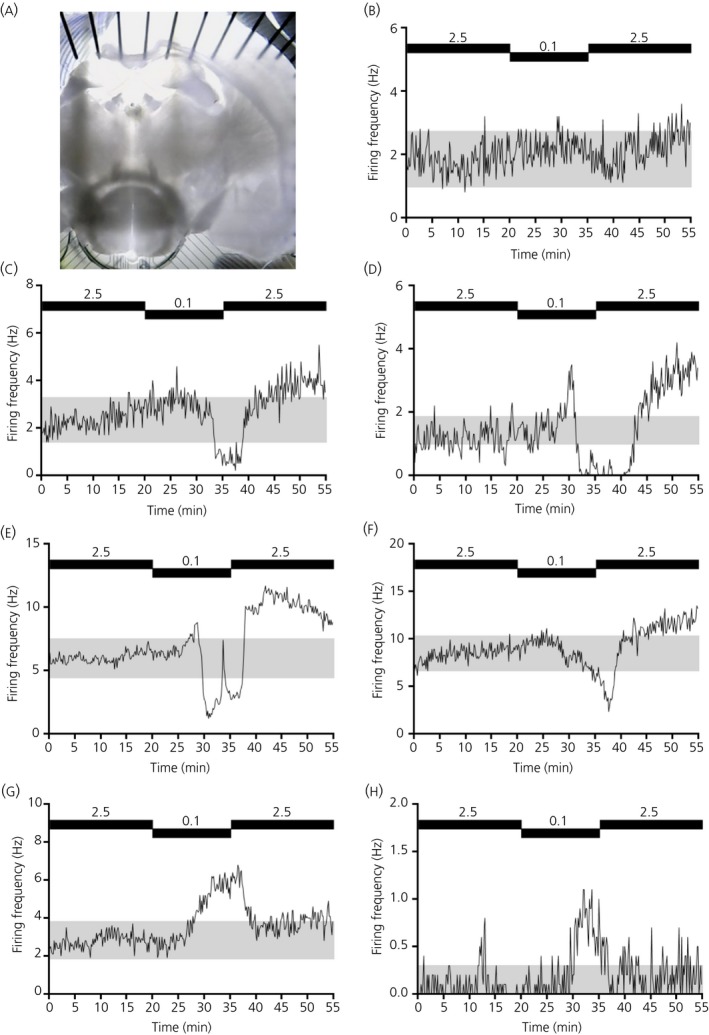
Representative examples of multi‐electrode array (MEA) captured multi‐unit activity across the ventromedial nucleus (VMN)‐containing slice. A, Representative image of an acute, coronal‐cut mouse VMN‐containing slice used for subsequent MEA recording (recording side shown). Representative perievent histograms from non‐responsive (B), glucose‐excited (glucose‐excited/presynaptically excited by an increase) (C‐E), late glucose‐excited/presynaptically excited by an increase responder (F) and glucose‐inhibited (glucose‐inhibited/presynaptically excited by a decrease) (G, H) multi‐unit channels. Black bars (B‐H) indicate bath application of 2.5 and 0.1 mmol L^‐1^ glucose (15 minutes). Grey shading (C‐H) represents the 95% confidence limits

Data in graphs and tables are expressed as the mean ± SE and/or median and interquartile range as appropriate. To compare groups, a paired *t* test (normally distributed data) or a Wilcoxon signed rank test (non‐normally distributed data) was used as appropriate. The Shapiro‐Wilk normality test was used to assess whether data were normally distributed. Two‐way ANOVA with repeated measures and Bonferroni post‐hoc test was used to evaluate the impact of glucose concentration on mean firing frequency between regions of the VMN. Statistical analyses were performed using prism (GraphPad Software Inc., San Diego, CA, USA).

### 2.7 Anatomical locations

Offline overlay of pMEA electrode sites and respective recorded slice images, with the aid of key landmarks (shape of the hippocampus, third ventricle, median eminence and location of the optic tracts) and reference to the Mouse Brain Atlas,^18^ were used to determine the location of each pMEA electrode site/channel: VMN, non‐VMN or non‐parenchymal (Table 1 [multi‐unit] and Table 1 [single‐unit] for 2.5 to 0.1 mmol L^‐1^ [40 minutes] studies). Specifically, the relative shape of both the hippocampus and third ventricle was studied to confirm the rostral‐caudal location of the mouse VMN‐containing slice (from bregma −1.22 mm up to –2.18 mm) and to verify that the cutting plane was not angled. The size and shape of the third ventricle then determined the optimal VMN‐containing brain slice(s) for recording, thus ensuring consistency between animals and experiments. Although every effort was made to only include electrodes in the analysis that fell within the VMN, recording locations are only approximate to the VMN and its subdivisions and there is a possibility that some electrode recordings are not from the VMN but, instead, from adjacent regions. Within the mouse hypothalamus, as a result of the challenges associated with accurate delineation of the sub‐regions, the VMN was split into a dorsomedial (DM) and ventrolateral (VL) region, with its central region equally split between the two regions. Collectively, this allowed for VMN regional differences to be studied and, importantly, to be distinguished from non‐VMN regions, with the latter being excluded from VMN‐related analyses (Table 2).

**Table 1:** Distribution of recording channels (multi‐unit) across the ventromedial nucleus (VMN) of the mouse hypothalamus

Slice	Total number of VMN channels	Total number of non‐VMN channels	Total number of non‐parenchymal channels	Number of DM‐VMN channels	Number of VL‐VMN channels
1	56	0	3	33	23
2	52	3	4	23	29
3	18	39	2	8	10
4	55	0	4	26	29
5	49	6	4	20	29
6	58	0	1	29	29
7	36	18	5	19	17
8	41	16	2	22	19
Total	365	82	25	180	185
Mean per slice	46	10	3	23	23
SEM	5	5	<1	3	3
Median per slice	51	5	4	23	26
IQR	40	<1	2	20	19

Note

Data rounded to the nearest integer. n = 5 mice (one or two slices per mouse).

Abbreviations: DM, dorsomedial; IQR, interquartile range; VL, ventrolateral.

**Table 2:** Distribution of total identified single‐units across the ventromedial nucleus (VMN) of the mouse hypothalamus

Slice	Total number of single‐units	Total number of VMN single‐units
1	6	6
2	2	2
3	5	5
4	12	12
5	9	8
6	5	5
7	12	6
8	10	8
Total	61	52
Mean per slice	8	7
SEM	1	1
Median per slice	8	6
IQR	5	5

Note

Data rounded to the nearest integer where appropriate. n = 5 mice (one or two slices per mouse).

Abbreviation: IQR, interquartile range.

## 3 RESULTS

### 3.1 Extracellular MEA recordings detect the presence of glucose‐sensing channels in the VMN

**Figure 2:**
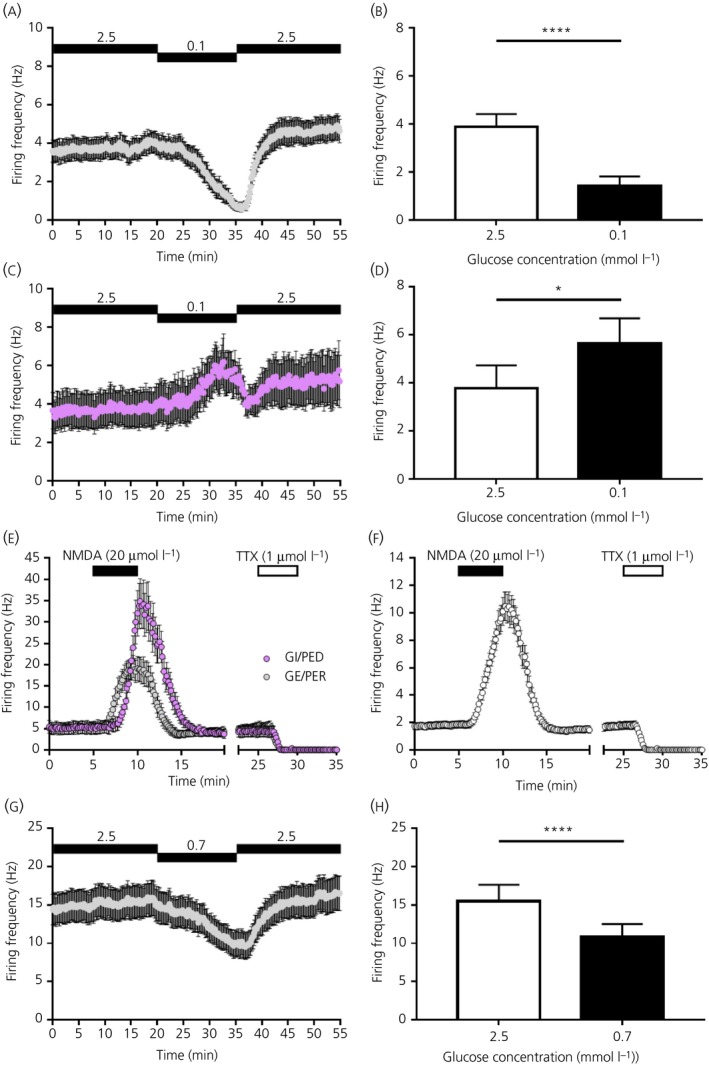
Firing frequency across the ventromedial nucleus (VMN) in response to changing glucose concentrations and to application of NMDA and tetrodotoxin (TTX) (multi‐unit analysis). A, Firing frequency over time in response to changing the extracellular glucose concentration from 2.5 to 0.1 mmol L^‐1^ (15 minutes) and back to 2.5 mmol L^‐1^ (indicated by black bars) across all VMN glucose‐excited (GE/presynaptically excited by an increase [PER]) channels. B, Mean firing frequency over the last 5 minutes in 2.5 mmol L^‐1^ glucose vs the last 5 minutes in 0.1 mmol L^‐1^ glucose across all VMN GE/PER channels. ****P < 0.0001 Wilcoxon matched‐pairs signed rank test, n = 31 GE/PER channels (four slices from four mice; total of 210 VMN channels). C, Firing frequency over time in response to changing the extracellular glucose concentration from 2.5 to 0.1 mmol L^‐1^ (15 minutes) and back to 2.5 mmol L^‐1^ (black bars) across all VMN glucose‐inhibited (GI/presynaptically excited by a decrease [PED]) channels. D, Mean firing frequency over the last 5 minutes in 2.5 mmol L^‐1^ glucose vs the last 5 minutes in 0.1 mmol L^‐1^ glucose across all VMN GI/PED channels. *P < 0.05, paired *t* test, n = 8 GI/PED channels (four slices from four mice; total of 210 VMN channels). E, Mean firing frequency over time across all VMN GE/PER and GI/PED channels in response to bath application (indicated by bars) of the glutamatergic NMDA receptor agonist, NMDA (20 µmol L^‐1^) and the voltage‐gated sodium channel blocker, TTX (1 µmol L^‐1^). Multi‐unit analysis of n = 28 and n = 8 NMDA responsive VMN GE/PER and GI/PED channels, respectively (four slices from four mice; total of 210 VMN channels). F, Mean firing frequency over time across all NMDA responsive VMN channels (including channels shown in E) in response to bath application (indicated by bars) of NMDA (20 µmol L^‐1^) and TTX (1 µmol L^‐1^). Multi‐unit analysis of a total of n = 163 NMDA responsive VMN channels (four slices from four mice; total of 210 VMN channels). G, Firing frequency over time in response to changing the extracellular glucose concentration from 2.5 to 0.7 mmol L^‐1^ (15 minutes) and back to 2.5 mmol L^‐1^ (indicated by black bars) across all VMN glucose‐excited (GE/PER) channels. H, Mean firing frequency over the last 5 minutes in 2.5 mmol L^‐1^ glucose vs the last 5 minutes in 0.7 mmol L^‐1^ glucose across all VMN GE/PER channels. ****P < 0.0001 Wilcoxon matched‐pairs signed rank test, n = 27 GE/PER channels (four slices from four mice; total of 215 VMN channels). Data are expressed as the mean ± SEM

To our knowledge, this is the first study to employ the MEA extracellular recording technique to investigate glucosensation in the VMN. Using this neurophysiological recording approach, we performed some preliminary experiments to test whether protocols similar to those used in patch clamp experiments could be employed to reveal the presence of glucose‐sensing channels in VMN‐containing brain slices prepared from mice (Figure 1A). By lowering the extracellular glucose concentration from 2.5 to 0.1 mmol L^‐1^ for 15 minutes, we found that, although most channels did not change their firing frequency (Figure 1B), approximately 15% (n = 31 out of 210 VMN channels: for pooled data, see Figure 2A,B) of VMN channels could be classified as GE/PER (Figure 1C‐E). The most commonly observed GE/PER channel response profiles are shown in Figure 1C,D (MUA analysis). Of note, a proportion of VMN channels were not included in any further analysis (~5%; n = 11 out of 210 VMN channels) because they were classified as late GE/PER responders (Figure 1F): despite the clear decline in firing rates during the 0.1 mmol L^‐1^ glucose application, firing rates did not cross the 95% confidence limits (grey shading) within the 15‐minute application window; thus, they were not classified as GE/PER channels. Nonetheless, as expected, GE/PER channels (as did late GE/PER responders) displayed a reversible and sustained reduction in firing frequency in response to a lowering of the extracellular glucose concentration from 2.5 to 0.1 mmol L^‐1^ (Figure 2A,B).

Although rarer, we also observed VMN channels that could be classified as GI/PED channels, accounting for < 4% (n = 8 out of 210 VMN channels: for pooled data, see Figure 2C,D) of VMN channels (Figure 1G,H, MUA analysis). By contrast to GE/PER channels, GI/PED channels displayed a reversible increase in firing frequency in response to a lowering of the extracellular glucose concentration from 2.5 to 0.1 mmol L^‐1^ for 15 minutes. GI/PED channels observed had similar characteristics to non‐adapting GI (Figure 1G) and adapting GI profiles (Figure 1H) as described previously^21^; however, we could not (in this or other experiments) stratify the data by the two GI channel subtypes with confidence, as a result of the low channel number.

As a positive control, the ionotropic glutamate (NMDA) receptor agonist NMDA was bath applied to confirm cell/slice responsiveness. All GI/PED (n = 8/8) and 90% of GE/PER (n = 28/31) VMN channels were NMDA responsive (Figure 2E). Indeed, approximately 78% of VMN channels (including those classified as glucose‐sensing) were NMDA responsive (n = 163 out of 210 VMN channels) (Figure 2F). The bath application of the voltage‐gated sodium channel blocker TTX completely silenced the recordings (Figure 2E,F), confirming that the acquired signals exclusively reflected sodium‐dependent action potentials. This finding also highlights one of the known advantages of the pMEA system: the high signal‐to‐noise ratio.^17^

We also found evidence of glucose‐sensing channels within the mouse VMN in response to less extreme changes in the extracellular glucose concentration: lowering the extracellular glucose concentration from 2.5 to 0.7 mmol L^‐1^ for 15 minutes (more closely reflecting brain glucose levels after a 24‐hour/overnight fast 1). With this experimental protocol, we found that, although most channels did not change their firing frequency, approximately 13% (n = 27 out of 215 VMN channels: for pooled data, see Figure 2G,H) of VMN channels could be classified as GE/PER. These GE/PER channels also displayed a reversible and sustained reduction in firing frequency in response to a lowering the of extracellular glucose concentration from 2.5 to 0.7 mmol L^‐1^ (Figure 2G,H). However, only one channel was classified as an adapting GI/PED channel with this protocol. Furthermore, with this protocol (ie, 2.5 to 0.7 mmol L^‐1^ glucose), there was again a proportion of channels (< 2%; n = 4 out of 215 VMN channels) resembling those previously classified as late GE/PER responders (Figure 1F).

In summary, although we found evidence of glucose‐sensing channels within the VMN with an extracellular recording method after a 15‐minute application of 0.1 mmol L^‐1^ glucose, for the remainder of the studies, an extended 40‐minute application was used. This was for two main reasons: (i) to circumvent the issue of omitting late GE/PER (and potentially GI/PED) responders and (ii) to eventually reach a steady‐state (ie, plateau) in the response on a population level. We chose the 40‐minute timepoint considering that it would likely produce the largest effect across multiple channels and slices, and accepting that some channels may change their frequency more slowly, which we would also capture using this methodology. As such, data discussed from this point onwards is in response to a lowering of the extracellular glucose concentration from 2.5 to 0.1 mmol L^‐1^ for 40 minutes.

### 3.2 Most glucose‐sensing channels observed in the VMN were glucose‐excited (GE/PER)

**Figure 3:**
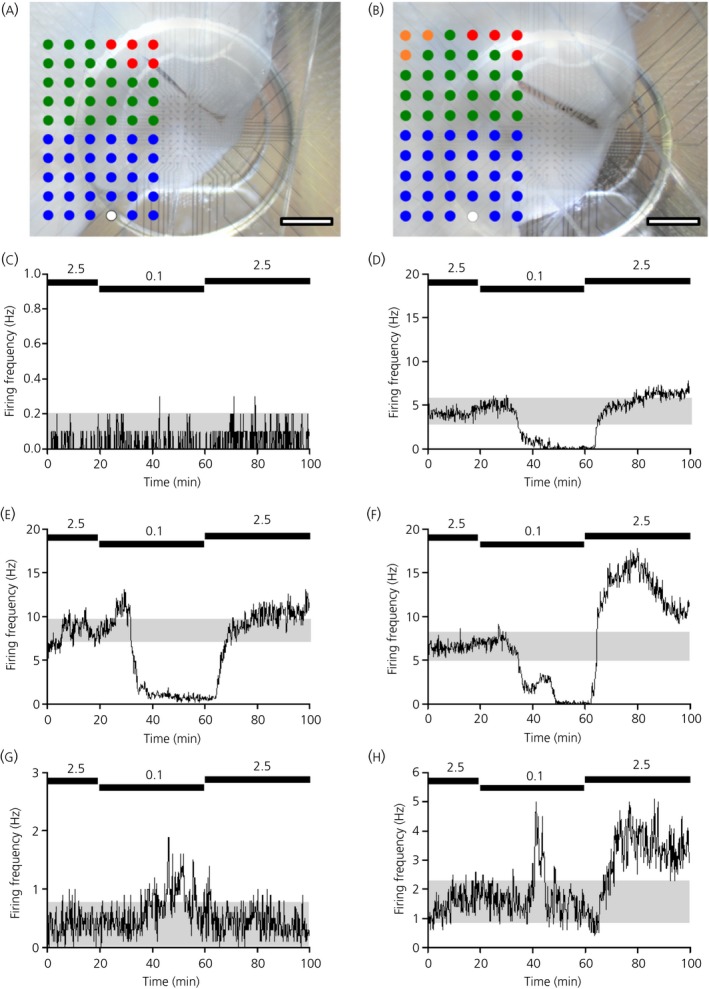
Representative examples of multi‐electrode array (MEA) channel location across the ventromedial nucleus (VMN)‐containing slice and multi‐unit perievent histograms. A, B, Representative overlaid images of VMN‐containing slices, indicating the location of the pMEA electrode sites/channels. Insert shows assigned anatomical location of each channel within the VMN. Green circles = dorsomedial VMN; blue circles = ventrolateral VMN; red circles = non‐parenchymal; orange circles = non‐VMN; open circle = reference electrode. Scale bars = 700 µm. Representative perievent histograms from non‐responsive (C), glucose‐excited (glucose‐excited/presynaptically excited by an increase) (D‐F) and glucose‐inhibited (glucose‐inhibited/presynaptically excited by a decrease) (G, H) multi‐unit channels. Black bars (C‐H) indicate bath application of 2.5 and 0.1 mmol L^‐1^ (40 minutes) glucose. Grey shading (C‐H) represents the 95% confidence limits

**Figure 4:**
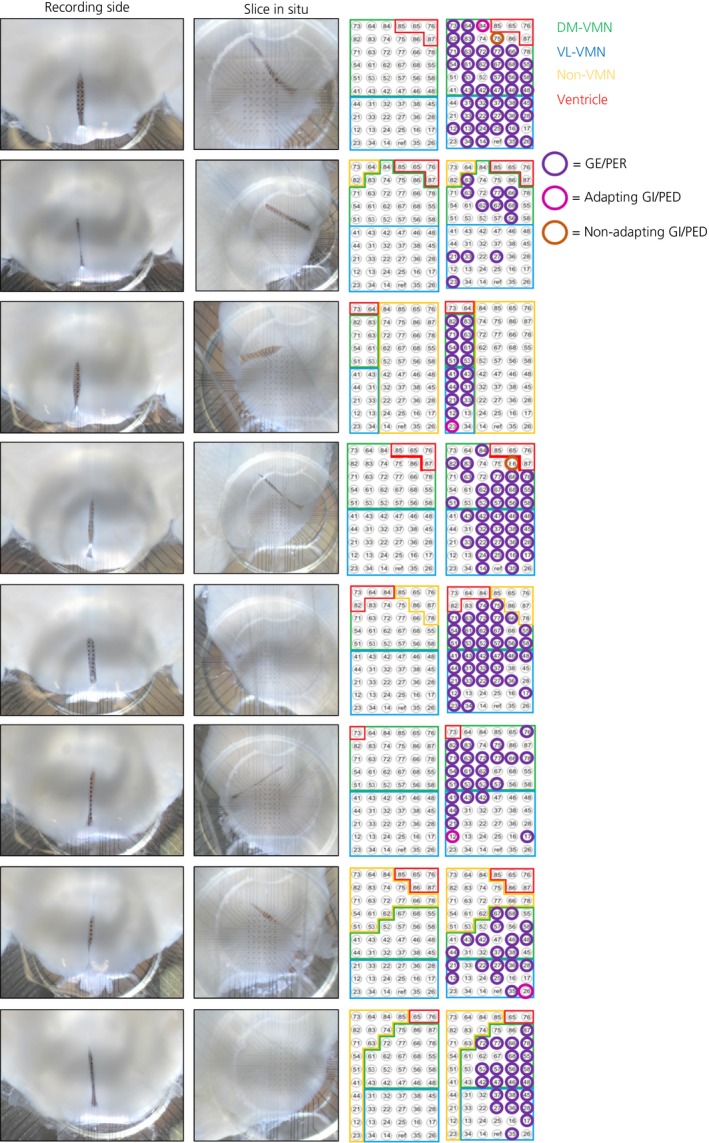
Multi‐electrode array (MEA) channel location and distribution of glucose‐sensing channels across all ventromedial nucleus (VMN)‐containing slices (40 minutes 0.1 mmol L^‐1^ glucose studies). Slices (thickness 300 µm) containing the medial portion of the mouse VMN were collected (bregma −1.22 mm up to −2.18 mm) and the recording side of each slice (panels on left) was placed recording side down onto a perforated MEA (pMEA) (see slice in situ panels). Note that the slice is held in place by negative pressure (vacuum‐generated suction is gently applied through perforations of the MEA), which may change the appearance of the slice in situ. The numbered grids (panels on the right) indicate location of the pMEA electrode sites/channels: green = dorsomedial (DM) VMN; blue = ventrolateral (VL) VMN; red = non‐parenchymal; yellow = non‐VMN. The location of the glucose‐excited (GE/presynaptically excited by an increase [PER]) channels (multi‐unit) on the arrays are indicated by open purple circles. The pink and orange open circles indicate the location of the glucose‐inhibited (GI/presynaptically excited by a decrease [PED]) adapting and non‐adapting GI channels, respectively (multi‐unit)

**Figure 5:**
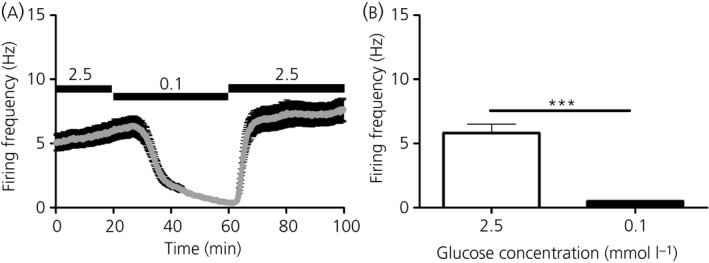
Firing frequency across the ventromedial nucleus (VMN) in response to changing glucose concentrations (multi‐unit analysis). A, Firing frequency over time in response to changing the extracellular glucose concentration from 2.5 to 0.1 mmol L^‐1^ (40 minutes) and back to 2.5 mmol L^‐1^ (indicated by black bars) across all VMN glucose‐excited (GE/presynaptically excited by an increase [PER]) channels. B, Mean firing frequency over the last 5 minutes in 2.5 mmol L^‐1^ glucose vs the last 5 minutes in 0.1 mmol L^‐1^ glucose across all VMN glucose‐excited (GE/PER) channels. Data are expressed as the mean ± SEM. ***P < 0.001 Wilcoxon matched‐pairs signed rank test, n = 211 GE/PER channels (eight slices from five mice)

Using the same pMEA neurophysiological recording approach (Figures 3A,B and 4) combined with the extended (40 minutes) bath application of 0.1 mmol L^‐1^ glucose, approximately 60% of glucose‐sensing channels (MUA analysis) detected across the VMN were classified as GE/PER (Figure 3D‐F and Table 3), with the most commonly observed GE/PER channel response profiles shown in Figure 3D,E (note the similarities to Figure 1C‐E). Although this value is higher than that observed with the previous (15 minutes) experimental protocol (potentially in part as a result of capture and inclusion of late GE/PED responders), these GE/PER channels still displayed a reversible and sustained reduction in firing frequency in response to a lowering of the extracellular glucose concentration from 2.5 to 0.1 mmol L^‐1^ for 40 minutes (Figure 5).

**Table 3:** Percentage of glucose‐sensing channels (multi‐unit), per slice, across the mouse ventromedial nucleus (VMN) of the hypothalamus

Region	Glucose‐excited (GE/PER) channels/slice (%, mean ± SEM)	Glucose‐inhibited (GI/PED) channels/slice (%, mean ± SEM)	Non‐responsive channels/slice (%, mean ± SEM)	Mean ± SEM channels/slice (n, total channels)
Total VMN	59.49 ± 7.21	1.71 ± 0.66	38.64 ± 7.67	46 ± 5 (365)
DM‐VMN	69.07 ± 7.07*	0.86 ± 0.57	30.10 ± 7.07*	23 ± 3 (180)
VL‐VMN	51.46 ± 8.78*	2.42 ± 1.33	46.31 ± 9.00*	23 ± 3 (185)

Note

Data from eight slices from five mice, one or two slices per mouse. Number of channels rounded to the nearest integer.

Abbreviations: DM, dorsomedial; VL, ventrolateral; GE, glucose‐excited; GI, glucose‐inhibited; PER, presynaptically excited by an increase; PED, presynaptically excited by a decrease.

* *P* < 0.05, Paired *t* test DM‐VMN vs VL‐VMN.

The mean number of GI/PED channels (MUA analysis) observed across the VMN was < 2% (Figure 3G,H and Table 3): a low percentage similar to that previously observed with the 15‐minute 0.1 mmol L^‐1^ glucose application (< 4%). GI/PED channels displayed a reversible increase in firing frequency in response to a lowering of the extracellular glucose concentration from 2.5 to 0.1 mmol L^‐1^ for 40 minutes. The GI/PED channels had characteristics similar to non‐adapting GI (Figure 3G) and adapting GI profiles (Figure 3H) as described previously.^21^ Rarely, GI/PED channels showed increased firing upon return to 2.5 mmol L^‐1^ glucose (Figure 3H). MUA analysis revealed a low basal firing rate of GI/PED channels, which was increased in response to a lowering of the extracellular glucose concentration from 2.5 to 0.1 mmol L^‐1^ for 40 minutes (mean ± SEM: 0.63 ± 0.25 Hz and 0.79 ± 0.38 Hz, respectively, n = 6 channels; data not shown). This increase in the mean neuronal firing rate is likely underestimated as a result of the transient responsiveness of adapting GI channels in low glucose.

### 3.3 More glucose‐excited (GE/PER) channels were found in the dorsomedial compared to the ventrolateral subdivision of the VMN

**Figure 6:**
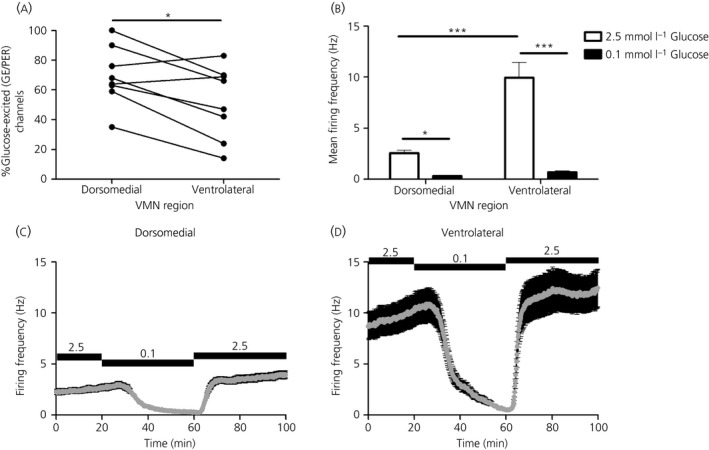
Regional differences in firing frequency of glucose‐excited (GE/presynaptically excited by an increase [PER]) channels across the ventromedial nucleus (VMN) (multi‐unit analysis). A, Percentage of glucose‐excited (GE/PER) channels in the dorsomedial compared with the ventrolateral region of the VMN; *P < 0.05, paired *t* test. B, Mean firing frequency over the last 5 minutes in 2.5 mmol L^‐1^ glucose vs the last 5 minutes in 0.1 mmol L^‐1^ glucose across all VMN GE/PER channels. Mean firing frequency over time in response to changing glucose concentrations from 2.5 to 0.1 mmol L^‐1^ (40 minutes) and back to 2.5 mmol L^‐1^ across GE/PER channels located in (C) the dorsomedial‐VMN (DM‐VMN) and (D) the ventrolateral‐VMN (VL‐VMN) region; Two‐way repeated measures ANOVA with Bonferroni post‐hoc: VMN region, *F*_VMN region_ _1,418_ = 32.95, ***P < 0.001; glucose convcentration, *F*_1,418_ = 72.57, ***P < 0.001; interaction, *F*_1,418_ = 26.78, ***P < 0.001; Bonferroni post‐hoc tests, *P < 0.05, ***P < 0.001. Data are expressed as the mean ± SEM. DM‐VMN, n = 119, VL‐VMN, n = 92 GE/PER channels (eight slices from five mice)

Within the VMN, the DM‐VMN had significantly more GE/PER channels than the VL‐VMN in response to a lowering of the extracellular glucose concentration from 2.5 to 0.1 mmol L^‐1^ for 40 minutes (Figures 6A and 4 and Table 3). There was no significant difference in the distribution of GI/PED channels between these VMN sub‐regions (Figure 4 and Table 3).

### 3.4 Glucose‐excited (GE/PER) channels in the ventrolateral portion of the VMN displayed a higher spontaneous basal firing frequency

**Figure 7:**
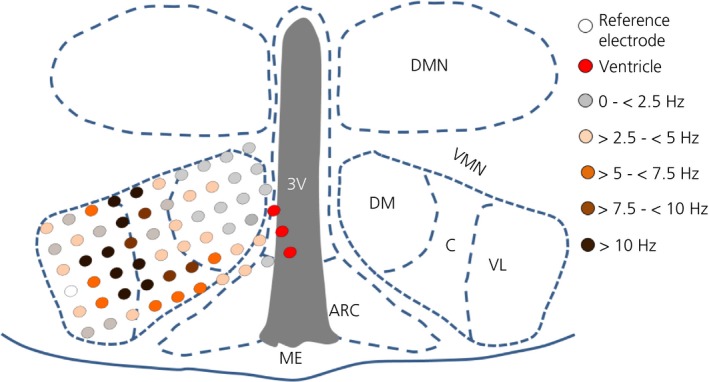
Representative heat map of the distribution of glucose‐excited (GE/presynaptically excited by an increase [PER]) channels across the mouse ventromedial nucleus (VMN) relative to their respective basal firing frequency in 2.5 mmol L^‐1^ glucose. Glucose‐excited (GE/PER) channels within the ventrolateral‐VMN (VL‐VMN) display a higher basal firing frequency compared to those in the dorsomedial‐VMN (DM‐VMN): data from 365 channels across eight slices from five mice (multi‐unit analysis). ME, median eminence; ARC, arcuate nucleus; 3V, third ventricle; C, central

The baseline firing frequency of GE/PER channels (ie, in 2.5 mmol L^‐1^ glucose; MUA analysis) was significantly higher in the VL‐VMN compared to the DM‐VMN region VMN in response to a lowering of the extracellular glucose concentration from 2.5 to 0.1 mmol L^‐1^ for 40 minutes (Figure 6B‐D). From these data, a representative heat map was generated in which the firing activity across the VMN was plotted relative to anatomical location: this analysis indicated that channels with the highest average basal firing frequency were located in the VL‐VMN proximal to the central region (Figure 7).

### 3.5 Glucose‐inhibited (GI/PED) channels were found across both subdivisions of the VMN

MUA analysis revealed a relatively low number of GI/PED channels (n = 6) in response to a lowering of the extracellular glucose concentration from 2.5 to 0.1 mmol L^‐1^ for 40 minutes. GI/PED channels were found in both the DM‐VMN and VL‐VMN region (Figure 4 and Table 3). However, as a result of the low number of GI/PED VMN channels detected overall, these observations should be interpreted with caution.

### 3.6 Analysis of the single‐unit data supported the observations of the multi‐unit analysis

The SUA supported the MUA data, with 60% of single‐units across the VMN being GE/PER (Table 4) and < 7% being GI/PED in response to a lowering of the extracellular glucose concentration from 2.5 to 0.1 mmol L^‐1^ for 40 minutes, with the latter being a higher percentage than was seen for the GI/PED MUA analysis. There were insufficient single‐units per slice within the VMN sub‐regions to provide meaningful analysis of the within VMN distribution.

**Table 4:** Percentage of glucose‐sensing single‐units, per slice, across the ventromedial nucleus (VMN) of the hypothalamus

Region	Glucose‐excited (GE/PER) single‐units/slice (%, mean ± SEM)	Glucose‐inhibited (GI/PED) single‐units/slice (%, mean ± SEM)	Non‐responsive single‐units/slice (%, mean ± SEM)	Mean ± SEM single‐units/slice (n, total channels)
Total VMN	60.17 ± 10.34	6.67 ± 3.27	33.21 ± 10.30	7.1 ± 1.29 (52)

Note

Data from 8 slices from 5 mice, 1‐2 slices/mouse. Number of channels rounded to the nearest integer.

Abbreviations: GE, glucose‐excited; GI, glucose‐inhibited; PER, presynaptically excited by an increase; PED, presynaptically excited by a decrease.

## 4 DISCUSSION

In the present study, we report that, using the pMEA recording technique in ex vivo brain slices, both multi‐unit and single‐unit analyses confirmed the presence of glucose‐sensing neurones across the dorsal‐ventral extent of the mouse VMN. This was observed in response to a lowering of the extracellular glucose concentration from 2.5 mmol L^‐1^ to either 0.1 or 0.7 mmol L^‐1^ glucose for 15 minutes and from 2.5 to 0.1 mmol L^‐1^ glucose for 40 minutes. On this occasion, we focused our main analysis on VMN‐containing slices that were exposed to 0.1 mmol L^‐1^ glucose for 40 minutes to enable the detection of the maximal number of GE/PER units and the achievement of a steady‐state in the response to low glucose at the population level.

In agreement with published data utilising single‐cell recording techniques in rodent VMN slices, GE/PER channels were much more prevalent than GI/PED channels.^10^, ^23^ At the multi‐unit (population) level, although significantly more GE/PER channels were found in the DM‐VMN, VL‐VMN GE/PER channels showed a significantly higher basal firing frequency (in 2.5 mmol L^‐1^ glucose) than DM‐VMN GE/PER channels. MUA and SUA analysis revealed that GI/PED channels are distributed across the subdivisions of the VMN. Further interpretation of the data should be made with caution, as a result of the relatively low number of GI/PED channel subtypes observed in the present study. It is of interest to note the relative quiescence of many of these GI/PED channels (and single‐units) during baseline recordings in 2.5 mmol L^‐1^ glucose, in line with findings from patch clamp studies.^7^

The proportion of glucose‐sensing channels across the VMN that has been reported previously in single‐cell neurophysiological studies in male rodents varies from 50% across the whole VMN 10 to 20%‐80% in the VL‐VMN.^21^, ^22^, ^23^ Data from the present study suggest that, across the whole VMN, the proportion of glucose‐sensing channels is 15%‐60% depending on the duration of the exposure to 0.1 mmol L^‐1^ glucose, which appears to be an important variable in determining the proportion of glucose‐sensing channels. Nonetheless, when single‐units were isolated from the multi‐unit data for the extended (40 minutes) 0.1 mmol L^‐1^ glucose studies, the high percentage of glucose‐sensing single‐units remains. Some of the reported differences between studies may also be attributed to the age of the animals at the time of study because many studies using single‐cell recordings were performed in brain slices from 14‐21‐day‐old juvenile animals, or indeed from species differences because many of the comparable studies have been performed in rats.^10^, ^23^, ^24^, ^25^ Indeed, separate studies have indicated that there are important species differences with respect to the percentage of glucose‐sensing neurones in the VL‐VMN: 20% in the juvenile male rat 23 and 80% in the juvenile male mouse.^21^

The regional differences in number and firing rate of the GE/PER channels observed in the mouse VMN in the present study may relate to differences in the anatomical location and inputs to the sub‐regions within the VMN.^26^ The higher number of GE/PER channels in the DM‐VMN may be a result of the proximity of this portion of the VMN to the third ventricle. Tanycytes lining the third ventricle have glucose‐sensing capacity and extend projections into the VMN,^27^ enabling direct communication of cerebrospinal fluid glucose levels to DM‐VMN neurones. DM‐VMN neurones are directly responsive to the key adipostatic hormone leptin via leptin‐receptors expressed in this region.^28^ In addition, the transcription factor steroidogenic‐factor 1 (SF‐1) is highly expressed in the DM‐ and central portion of the VMN of the adult mouse,^29^ and deletion of leptin‐receptors from SF‐1 neurones in mice using a conditional transgenic knockout approach results in obesity.^28^, ^30^, ^31^ Interestingly, studies in rodents have shown that infusion or microinjection of leptin directly in to the VMH enhances insulin sensitivity and increases glucose uptake in peripheral tissues, including skeletal muscle, heart and interscapular brown adipose tissue 32, 33: these effects may be driven by a leptin‐activated subset of SF‐1 DM‐VMN GE neurones.^34^ In line with these findings, chemogenetic activation of SF‐1 VMN neurones stimulates interscapular brown adipose tissue, heart and red‐type skeletal muscle glucose uptake.^35^ Of note, optogenetic modulation of SF‐1 VMN neurones also implicates these cells in the regulation of systemic glycaemia, with direct stimulation promoting hyperglycaemia, whereas direct inhibition of these neurones attenuates the counter‐regulatory response to insulin‐induced hypoglycaemia.^36^ Collectively, such observations suggest that specific subsets of SF‐1 VMN neurones may play distinct roles in systemic glucoregulation. Anatomical tracing studies indicate that the SF‐1 VMN neurones critical for the regulation of glycaemia receive inputs from the lateral parabrachial nucleus and mediate their effect on glucose homeostasis via projections to the anterior portion of the bed nucleus of the stria terminalis (aBNST).^36^ This projection pattern of SF‐1 VMN neurones is supported by work conducted by an independent group demonstrating projections from these neurones to sites implicated in regulating sympathetic and autonomic activity,^37^ including the periaqueductal grey, aBNST, lateral parabrachial nucleus, nucleus of the solitary tract and rostral ventrolateral medulla. Taken together, these findings implicate neurones in the DM‐VMN in the central regulation of systemic glycaemia, which may explain the relative abundance of GE neurones in this region of the VMN.

In the present study, the GE/PER channels that showed the highest firing rate in 2.5 mmol L^‐1^ glucose were located in the VL‐VMN: the heat map indicates their proximity to the central portion of the VMN. This finding is in agreement with published studies indicating that GE neurones are enriched in the VL‐VMN.^23^ The central and VL‐VMN contain high levels of brain‐derived neurotrophic factor, 38 as well as demonstrating expression of neuroendocrine receptors including oestrogen‐receptor α 39, progesterone‐receptors,^40^ androgen receptors^41^ and oxytocin receptors.^42^, ^43^ Indeed, although the relative percentage of VL‐VMN glucose‐sensing neurones is reported to be similar in male and female mice, the proportion of the different subtypes of glucose‐sensing neurones in the VL‐VMN (GE, adapting GI and non‐adapting GI) appears to be sexually dimorphic, which may relate to differential activation of sex steroid hormone receptors impacting neuronal glucose‐sensing.^21^, ^22^ Furthermore, the VL‐VMN is strongly implicated in mediating sexual behaviour and aggression.^44^ It is not clear why the GE/PER channels in this region showed higher basal firing rates in response to 2.5 mmol L^‐1^ glucose; one possibility could be that, because both sexual behaviour and aggression are energetically expensive, it may make biological sense that a key component in the circuit controlling these behaviours is sensitive to changes in systemic energy availability. Although previous studies have performed detailed electrophysiological characterisation of VMN neurones,^45^ to our knowledge, this is the first report of observed regional differences in basal firing frequency in 2.5 mmol L^‐1^ glucose across the VMN, highlighting a strength of simultaneous recording across the slice, which the MEA approach enables.

The proportion of GI/PED channels detected across the pMEA sites (< 2% MUA, < 7 SUA%; 40 minutes 0.1 mmol L^‐1^ glucose) were lower than that reported in previously published studies (single‐cell recordings) across the VMN from rats,^10^ or the VL‐VMN from mice.^21^, ^22^ However, our observations were higher than the 0.4% GI neurones reported in the rat VL‐VMN.^23^ As such, there appears to be variability in the reported frequency of GI neurones in the VMN. It is possible that, using our extracellular recording method, the prevalence of GI/PED channels is artificially lower than expected because the activity may be masked by the dominating activity of neighbouring GE neurones. This may explain the response profile where we occasionally observed a hump in the firing rate of GE/PER channels during bath application of 0.1 mmol L^‐1^ glucose; for example, during the first 10‐15 minutes of low glucose perfusion (Figure 1D at 30 minutes and Figure 3E at 20‐30 minutes) or during the nadir phase (Figure 1E at 30‐35 minutes and Figure 3F at 40‐50 minutes). Nevertheless, in the present study, the percentage of GI/PED channels across the VMN was significantly lower than that of GE/PER channels.

GI neurones in the VMH that specifically express neuronal nitric oxide synthase have been shown to be important for both neuronal glucosensing and regulation of the counter‐regulatory response to hypoglycaemia in vivo.^46^ Furthermore, VMN GE and GI neurones have been shown to express SF‐1.^47^ A subpopulation of VMN SF‐1 neurones has been shown to co‐localise with pituitary adenylate cyclase‐activating peptide (PACAP) 48 and recent work has identified a population of intrinsically GI VMN PACAP‐expressing neurones.^9^ Although comprising a relatively small neuronal population, they display a wide distribution across the VMN and are considered to play a role in systemic glucose regulation because chemogenetic stimulation of these neurones in vivo resulted in inhibition of insulin secretion, which led to a reduced glucose tolerance.^9^ Interestingly, GI VMN PACAP‐expressing neurones (15% of which were found to be neuronal nitric oxide synthase‐positive) send projections both within the VMH and to other brain areas, including the paraventricular nucleus, lateral hypothalamus, aBNST, paraventricular nucleus of the thalamus and periaqueductal grey,^9^ in line with the findings reported by Meek *et al*.^36^

In summary, in the present study, we have utilised the pMEA recording technique to provide an unbiased assessment of the percentage and distribution of glucose‐sensing neurones across the mouse VMN (ex vivo). Although the pMEA method cannot provide finer, more detailed information regarding the neurophysiological properties of cells, such as can be obtained by the patch clamp technique, it is still a very useful neurophysiological tool for examining both population and single‐cell level responses simultaneously across brain nuclei, thus providing, using a high‐throughput approach, key functional neuroanatomical information that could complement and inform the design of future single‐cell studies. In practical terms, MEA recordings are arguably more user‐friendly and technically less demanding to perform than the gold standard patch clamp method and, at the same time, offer a high spatial and temporal resolution.^14^ Finally, the in vitro MEA method permits cell‐non‐invasive, long‐term, stable recordings to be performed, and, in combination with optogenetic, chemogenetic, pharmacological and/or calcium imaging approaches, this method can be used to refine our understanding of other neuroendocrine networks.

## CONFLICT OF INTERESTS

The authors declare that they have no conflicts of interest.

## Data Availability

The data that support the findings of this study are available from the corresponding author upon reasonable request.

## References

[1] Routh VH , Hao L , Santiago AM , Sheng Z , Zhou C . Hypothalamic glucose sensing: making ends meet. Front Syst Neurosci. 2014;8:236. 25540613 10.3389/fnsys.2014.00236 PMC4261699

[2] de Vries MG , Arseneau LM , Lawson ME , Beverly JL . Extracellular glucose in rat ventromedial hypothalamus during acute and recurrent hypoglycemia. Diabetes. 2003;52:2767‐2773. 14578295 10.2337/diabetes.52.11.2767

[3] Dunn‐Meynell AA , Sanders NM , Compton D , et al. Relationship among brain and blood glucose levels and spontaneous and glucoprivic feeding. J Neurosci. 2009;29:7015‐7022. 19474328 10.1523/JNEUROSCI.0334-09.2009 PMC2728115

[4] Silver IA , Erecinska M . Extracellular glucose concentration in mammalian brain: continuous monitoring of changes during increased neuronal activity and upon limitation in oxygen supply in normo‐, hypo‐, and hyperglycemic animals. J Neurosci. 1994;14:5068‐5076. 8046468 10.1523/JNEUROSCI.14-08-05068.1994 PMC6577171

[5] Karnani M , Burdakov D . Multiple hypothalamic circuits sense and regulate glucose levels. Am J Physiol Regul Integr Comp Physiol. 2011;300:R47‐55. 21048078 10.1152/ajpregu.00527.2010 PMC3023280

[6] Routh VH . Glucose sensing neurons in the ventromedial hypothalamus. Sensors. 2010;10:9002‐9025. 22022208 10.3390/s101009002 PMC3196991

[7] Routh VH . Glucose‐sensing neurons: are they physiologically relevant? Physiol Behav. 2002;76:403‐413. 12117577 10.1016/s0031-9384(02)00761-8

[8] Burdakov D , Gerasimenko O , Verkhratsky A . Physiological changes in glucose differentially modulate the excitability of hypothalamic melanin‐concentrating hormone and orexin neurons in situ. J Neurosci. 2005;25:2429‐2433. 15745970 10.1523/JNEUROSCI.4925-04.2005 PMC6726089

[9] Khodai T , Nunn N , Worth AA , et al. PACAP neurons in the ventromedial hypothalamic nucleus are glucose inhibited and their selective activation induces hyperglycaemia. Front Endocrinol. 2018;9:632. 10.3389/fendo.2018.00632 PMC6218416 30425681

[10] Song Z , Levin BE , McArdle JJ , Bakhos N , Routh VH . Convergence of pre‐ and postsynaptic influences on glucosensing neurons in the ventromedial hypothalamic nucleus. Diabetes. 2001;50:2673‐2681. 11723049 10.2337/diabetes.50.12.2673

[11] Vazirani RP , Fioramonti X , Routh VH . Membrane potential dye imaging of ventromedial hypothalamus neurons from adult mice to study glucose sensing. J Vis Exp. 2013 10.3791/50861. PMC3992114 24326343

[12] Obien ME , Deligkaris K , Bullmann T , Bakkum DJ , Frey U . Revealing neuronal function through microelectrode array recordings. Front Neurosci. 2014;8:423. 25610364 10.3389/fnins.2014.00423 PMC4285113

[13] McClellan KM , Parker KL , Tobet S . Development of the ventromedial nucleus of the hypothalamus. Front Neuroendocrinol. 2006;27:193‐209. 16603233 10.1016/j.yfrne.2006.02.002

[14] Shaban H , O'Connor R , Ovsepian SV , Dinan TG , Cryan JF , Schellekens H . Electrophysiological approaches to unravel the neurobiological basis of appetite and satiety: use of the multielectrode array as a screening strategy. Drug Discov Today. 2017;22:31‐42. 27634341 10.1016/j.drudis.2016.09.003

[15] Lewicki MS . A review of methods for spike sorting: the detection and classification of neural action potentials. Network. 1998;9:R53‐78. 10221571

[16] Hanna L , Walmsley L , Pienaar A , Howarth M , Brown TM . Geniculohypothalamic GABAergic projections gate suprachiasmatic nucleus responses to retinal input. J Physiol. 2017;595:3621‐3649. 28217893 10.1113/JP273850 PMC5451736

[17] Reinhard K , Tikidji‐Hamburyan A , Seitter H , et al. Step‐by‐step instructions for retina recordings with perforated multi electrode arrays. PLoS ONE. 2014;9:e106148. 25165854 10.1371/journal.pone.0106148 PMC4148441

[18] Paxinos G , Franklin KBJ . The Mouse Brain in Stereotaxic Coordinates, 2nd edn Cambridge, MA: Academic Press; 2001.

[19] Walmsley L , Hanna L , Mouland J , et al. Colour as a signal for entraining the mammalian circadian clock. PLoS Biol. 2015;13:e1002127. 25884537 10.1371/journal.pbio.1002127 PMC4401556

[20] Booth C , Wayman CP , Jackson VM . An ex vivo multi‐electrode approach to evaluate endogenous hormones and receptor subtype pharmacology on evoked and spontaneous neuronal activity within the ventromedial hypothalamus; translation from female receptivity. J Sex Med. 2010;7:2411‐2423. 20487238 10.1111/j.1743-6109.2010.01843.x

[21] Santiago AM , Clegg DJ , Routh VH . Estrogens modulate ventrolateral ventromedial hypothalamic glucose‐inhibited neurons. Mol Metab. 2016;5:823‐833. 27688996 10.1016/j.molmet.2016.08.002 PMC5034617

[22] Santiago AM , Clegg DJ , Routh VH . Ventromedial hypothalamic glucose sensing and glucose homeostasis vary throughout the estrous cycle. Physiol Behav. 2016;167:248‐254. 27666162 10.1016/j.physbeh.2016.09.021 PMC5159237

[23] Cotero VE , Routh VH . Insulin blunts the response of glucose‐excited neurons in the ventrolateral‐ventromedial hypothalamic nucleus to decreased glucose. Am J Physiol Endocrinol Metab. 2009;296:E1101‐E1109. 19223652 10.1152/ajpendo.90932.2008 PMC2681311

[24] Song Z , Routh VH . Differential effects of glucose and lactate on glucosensing neurons in the ventromedial hypothalamic nucleus. Diabetes. 2005;54:15‐22. 15616006 10.2337/diabetes.54.1.15

[25] Song Z , Routh VH . Recurrent hypoglycemia reduces the glucose sensitivity of glucose‐inhibited neurons in the ventromedial hypothalamus nucleus. Am J Physiol Regul Integr Comp Physiol. 2006;291:R1283‐R1287. 16793940 10.1152/ajpregu.00148.2006

[26] Thompson RH , Swanson LW . Structural characterization of a hypothalamic visceromotor pattern generator network. Brain Res Brain Res Rev. 2003;41:153‐202. 12663080 10.1016/s0165-0173(02)00232-1

[27] Bolborea M , Dale N . Hypothalamic tanycytes: potential roles in the control of feeding and energy balance. Trends Neurosci. 2013;36:91‐100. 23332797 10.1016/j.tins.2012.12.008 PMC3605593

[28] Dhillon H , Zigman JM , Ye C , et al. Leptin directly activates SF1 neurons in the VMH, and this action by leptin is required for normal body‐weight homeostasis. Neuron. 2006;49:191‐203. 16423694 10.1016/j.neuron.2005.12.021

[29] Cheung CC , Kurrasch DM , Liang JK , Ingraham HA . Genetic labeling of steroidogenic factor‐1 (SF‐1) neurons in mice reveals ventromedial nucleus of the hypothalamus (VMH) circuitry beginning at neurogenesis and development of a separate non‐SF‐1 neuronal cluster in the ventrolateral VMH. J Comp Neurol. 2013;521:1268‐1288. 22987798 10.1002/cne.23226 PMC4304766

[30] Bingham NC , Anderson KK , Reuter AL , Stallings NR , Parker KL . Selective loss of leptin receptors in the ventromedial hypothalamic nucleus results in increased adiposity and a metabolic syndrome. Endocrinology. 2008;149:2138‐2148. 18258679 10.1210/en.2007-1200 PMC2329259

[31] Viskaitis P , Irvine EE , Smith MA , et al. Modulation of SF1 neuron activity coordinately regulates both feeding behavior and associated emotional states. Cell Rep. 2017;21:3559‐3572. 29262334 10.1016/j.celrep.2017.11.089 PMC5746599

[32] Kamohara S , Burcelin R , Halaas JL , Friedman JM , Charron MJ . Acute stimulation of glucose metabolism in mice by leptin treatment. Nature. 1997;389:374‐377. 9311777 10.1038/38717

[33] Minokoshi Y , Haque MS , Shimazu T . Microinjection of leptin into the ventromedial hypothalamus increases glucose uptake in peripheral tissues in rats. Diabetes. 1999;48:287‐291. 10334303 10.2337/diabetes.48.2.287

[34] Shimazu T , Minokoshi Y . Systemic Glucoregulation by Glucose‐Sensing Neurons in the Ventromedial Hypothalamic Nucleus (VMH). J Endocr Soc. 2017;1:449‐459. 29264500 10.1210/js.2016-1104 PMC5686683

[35] Coutinho EA , Okamoto S , Ishikawa AW , et al. Activation of SF1 neurons in the ventromedial hypothalamus by DREADD technology increases insulin sensitivity in peripheral tissues. Diabetes. 2017;66:2372‐2386. 28673934 10.2337/db16-1344

[36] Meek TH , Nelson JT , Matsen ME , et al. Functional identification of a neurocircuit regulating blood glucose. Proc Natl Acad Sci USA. 2016;113:E2073‐E2082. 27001850 10.1073/pnas.1521160113 PMC4833243

[37] Lindberg D , Chen P , Li C . Conditional viral tracing reveals that steroidogenic factor 1‐positive neurons of the dorsomedial subdivision of the ventromedial hypothalamus project to autonomic centers of the hypothalamus and hindbrain. J Comp Neurol. 2013;521:3167‐3190. 23696474 10.1002/cne.23338

[38] Xu B , Goulding EH , Zang K , et al. Brain‐derived neurotrophic factor regulates energy balance downstream of melanocortin‐4 receptor. Nat Neurosci. 2003;6:736‐742. 12796784 10.1038/nn1073 PMC2710100

[39] Musatov S , Chen W , Pfaff DW , et al. Silencing of estrogen receptor alpha in the ventromedial nucleus of hypothalamus leads to metabolic syndrome. Proc Natl Acad Sci USA. 2007;104:2501‐2506. 17284595 10.1073/pnas.0610787104 PMC1892990

[40] Quadros PS , Wagner CK . Regulation of progesterone receptor expression by estradiol is dependent on age, sex and region in the rat brain. Endocrinology. 2008;149:3054‐3061. 18308846 10.1210/en.2007-1133 PMC2408808

[41] Simerly RB , Chang C , Muramatsu M , Swanson LW . Distribution of androgen and estrogen receptor mRNA‐containing cells in the rat brain: an in situ hybridization study. J Comp Neurol. 1990;294:76‐95. 2324335 10.1002/cne.902940107

[42] Bale TL , Pedersen CA , Dorsa DM . CNS oxytocin receptor mRNA expression and regulation by gonadal steroids. Adv Exp Med Biol. 1995;395:269‐280. 8713977

[43] Young LJ , Muns S , Wang Z , Insel TR . Changes in oxytocin receptor mRNA in rat brain during pregnancy and the effects of estrogen and interleukin‐6. J Neuroendocrinol. 1997;9:859‐865. 9419837 10.1046/j.1365-2826.1997.00654.x

[44] Hashikawa Y , Hashikawa K , Falkner AL , Lin D . Ventromedial hypothalamus and the generation of aggression. Front Syst Neurosci. 2017;11:94. 29375329 10.3389/fnsys.2017.00094 PMC5770748

[45] Sabatier N , Leng G . Spontaneous discharge characteristic of neurons in the ventromedial nucleus of the rat hypothalamus in vivo. Eur J Neurosci. 2008;28:693‐706. 18671740 10.1111/j.1460-9568.2008.06389.x

[46] Fioramonti X , Marsollier N , Song Z , et al. Ventromedial hypothalamic nitric oxide production is necessary for hypoglycemia detection and counterregulation. Diabetes. 2010;59:519‐528. 19934009 10.2337/db09-0421 PMC2809968

[47] Toda C , Kim JD , Impellizzeri D , Cuzzocrea S , Liu ZW , Diano S . UCP2 regulates mitochondrial fission and ventromedial nucleus control of glucose responsiveness. Cell. 2016;164:872‐883. 26919426 10.1016/j.cell.2016.02.010 PMC4770556

[48] Hawke Z , Ivanov TR , Bechtold DA , Dhillon H , Lowell BB , Luckman SM . PACAP neurons in the hypothalamic ventromedial nucleus are targets of central leptin signaling. J Neurosci. 2009;29:14828‐14835. 19940178 10.1523/JNEUROSCI.1526-09.2009 PMC6666015

